# The Begging Strategy of Andean Dogs: An Exploratory Study

**DOI:** 10.3390/ani13040704

**Published:** 2023-02-17

**Authors:** Alessandro Finzi, Eleonora Rava, Biagio D’Aniello

**Affiliations:** 1 Didactic and Research Centre for Rabbit Welfare and Production, 56019 Pisa, Italy; 2 School of History, University of St Andrews, St Andrews KY16 9AJ, UK; 3 Department of Biology, University of Naples Federico II, 80126 Naples, Italy

**Keywords:** dogs, begging behavior, animal cognition, begging strategy

## Abstract

**Simple Summary:**

In this study, we report particular begging strategies by Andean dogs and by humans on the unsurfaced road between the villages of Parotani and Cahiuasi in Bolivia, recording their position and behavioral displays. Begging locations, for both dogs and humans, were permuted with a score, according to the higher probability of receiving food. The occurrences of the correct positioning at the external and internal parts of the bend were compared using a statistical test. The dogs were always observed to lie down at the border of the road, mainly alone and where the hairpin bends had been formed because of the strong sloping, forcing vehicles to slow down. Humans were observed mainly in groups. The percentage of dogs lying down on the external parts of bends was 81.2%, while humans were observed at external bends in 63.6% of cases. The mean score of dogs was significantly higher than that of humans.

**Abstract:**

In this study, we report a particular begging strategy by Andean dogs and by humans on the unsurfaced road between the villages of Parotani and Cahiuasi in Bolivia. The positions of the dogs and humans begging and their behavioral displays were recorded. In dogs, the distance from each other was also recorded. Begging locations, for both dogs and humans, were permuted with a score, according to the higher probability of receiving food. The highest scores were assigned to the positions where cars had to slow down and the subjects were well visible, thus meriting a score corresponding to the higher probability of receiving a treat. The occurrences of the correct positioning at the external and internal parts of the bend were compared by a chi-square test. On a range of 93.3 km, the dogs were observed to always lie down at the border of the road, mainly alone (96.3%) and on hairpin bends, present due to the steep slope, obliging the vehicles to slow down. Humans were observed mainly in groups. The percentage of dogs lying on the external part of the bends was 81.2%, which was above the level of chance (*p* < 0.01). Humans were observed at external bends in 63.6% of cases, which was at chance level. Begging locations, for both dogs and humans, were permuted with a score according to the higher probability of receiving food. The mean scores were 1.48 and 0.65 for dogs and humans, respectively, and the difference was highly significant (*p* < 0.001).

## 1. Introduction

Begging behavior is a very common strategy used to obtain resources by conspecifics. It is an important part of the behavioral repertoire of the young of altricial species, in which offspring are highly dependent on parental care [1,2,3,4], but it is also widespread across adult vertebrates, such as birds [5] and mammals [6,7].

Begging may also be an interspecies signal, with humans targeted by several captive and wild species. Well-known are the “begging burros” of Custer State Park in the United States [8] and the wild elephants in Udawalawe National Park in Sri Lanka [9]. Furthermore, animals living in zoos often beg for food from visitors [10,11].

Although begging behavior is genetically codified as an instinctive behavior, learning plays an important role. For example, Asian Short-Clawed Otters (*Aonyx cinereus*), which are not fed by visitors, only start begging when they see a person approaching wearing the same color as caregivers [12], which underlines the discriminative learning of the potential source of food. Bottlenose dolphins (*Tursiops truncatus*) acquire their begging behavior toward humans through social learning, as they are more likely to beg when they have the opportunity to observe other subjects accepting food from humans [13]. In this study, we report a particular begging strategy that has been self-acquired by free-ranging Andean dogs.

The domestication process has undoubtedly genetically modified the interactive methods of dogs, allowing them to communicate effectively with humans [14,15]. Two hypotheses have tried to explain how artificial selection has affected dogs’ social skills. The by-product hypothesis [16] states that dogs’ socio-cognitive skills were acquired as a by-product of the selection for tameness; while the adaptation hypothesis considers that dogs could have been specifically selected according to their ability to use human communicative signals [17]. Reviewing all research on ostensive cues, such as eye contact and communicative gestures, some authors have come to the conclusion that the adaptation hypothesis is more suitable [18,19]. However, learning to use certain forms of instinctive communication in specific contexts is crucial for improving socio-communicative skills [20,21,22]. Indeed, the use of gazing behavior directed at humans [23,24,25] and the ability to follow human pointing gestures [26] appear strongly conditioned by ontogenesis. It is believed that begging toward humans is a natural behavior, which, once reinforced, will be performed more and more, although there are no scientific reports on the matter. Thus, while the tendency to beg is an innate behavior in dogs, the begging strategy chosen is one that can be learned and refined. Dogs can use a wide spectrum of begging behaviors toward humans, which range from active strategies, including behaviors aimed strongly at drawing the attention of the interlocutor (i.e., barking, pawing, whining), to more gentle approaches using passive strategies, such as gazing at the desired target and waiting for the potential help of a person. This means that the begging of dogs is a very flexible behavior and can be easily adapted to the environmental context. 

Along the winding Interandina road from Quillacollo to Oruro in Bolivia, at a height of 3000 m or more, it is possible to observe dogs along the road, still and lonely. Our scientific curiosity was sparked by a quote from the Bolivian writer Néstor Taboada Terán. In one of his books, Terán described people throwing chunks of bread from cars or buses to dogs and coins to the Indios along this road [27]. The quote excerpted from the book underlined an interesting begging behavior by humans and dogs in the same place and, possibly, with two different strategies. This stimulated our scientific curiosity, whereby the current research aimed at studying the distribution, location, and behavior of dogs and humans to understand the actual manifestations of their begging strategies. We are aware that a balanced or controlled study design cannot be performed due to the different goals of the two species: dogs were looking for an immediate need (i.e., food), while humans were asking for money. Thus, we are presenting an observational and exploratory study with some comparison between the begging strategies of dogs and humans.

## 2. Materials and Methods

The study design was observational with a convenience sample. It was performed in the year 1995 along the unsurfaced road between the villages of Parotani and Cahiuasi in Bolivia, with a range of 70.0 km to 163.3 km. We applied a classical linear transect used in ecological studies while traveling back and forth along the road in the morning and the afternoon, respectively, and recording all the dogs and humans begging. The latter generally appeared to be children and/or old people. Where the mountainside becomes very steep, the road presents many bends, forcing the cars to slow down. We recorded the position of the dogs and humans in relation to the bends and their behavioral displays. The distance between dogs was also recorded.

Begging locations, for both dogs and humans, were recorded with a score according to the higher probability of receiving food. The highest scores were assigned to the positions where cars had to slow down, and the subjects were well visible (Table 1).

When there were multiple begging subjects in the same place, any gains had to be shared between them, and the score was divided according to the number of subjects present. The occurrences of positioning at the external and internal parts of the bends were compared using a chi-square test, which was also applied to compare the scores of dogs and humans.

## 3. Results

**Figure 1:**
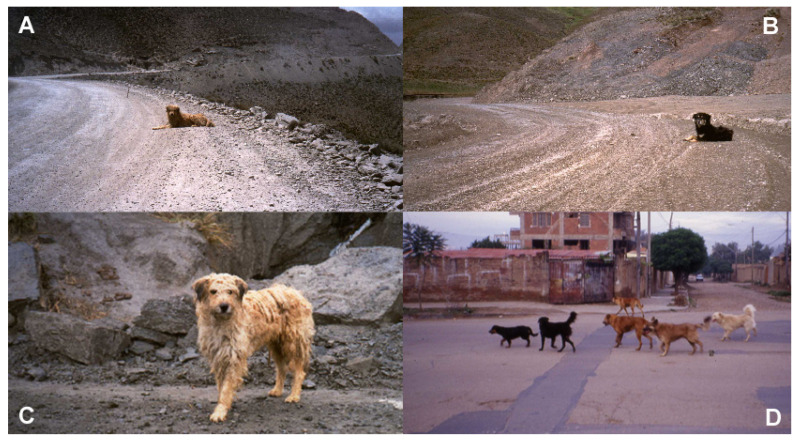
(**A**,**B**): Dog waiting at the external bend of the road where the vehicles must slow down. Note the dog’s state of attention once it became aware of our approaching car. (**C**) A typical Andean dog. (**D**) Dogs observed on the road gather in packs again when they return to the villages in the late afternoon.

Along the road, the dogs mostly lay down at the border of the road and by themselves (96.3%). Furthermore, dogs chose the hairpin bends, where, because of the steep slope, the vehicles slow down considerably (Figure 1A,B). In a few cases, dogs were standing (Figure 1C). Some of the dogs seen on the road were observed gathering in villages in the late afternoon (Figure 1D).

In comparison with dogs, humans were never alone and preferred to use active begging strategies, such as running after the lorries and cars.

The frequency of dogs in the morning was one every 2332 m as a mean average, but for the stretch of road between 116.0 km and 139.1 km, where bends are more frequent, dogs were observed at each hairpin bend at increasingly shorter distances from each other (962.5 m). On the way back, in the early afternoon, more dogs and humans were recorded (Table 2). 

The increase in the number of subjects observed in the afternoon was 89.7% and 80.9% for dogs and humans, respectively. The mean frequency of dogs was every 1775 m. However, in steeper places where the bends were closer together, dogs were observed every 250 m.

The percentage of dogs lying down on the external parts of the bends was 81.2%, which was above the chance level (*p* < 0.01). Humans were observed in external bends in 63.6% of cases, which was at a chance level.

When the number of wrong positions was compared with the total number of blind bends, the percentages were 8.7 % and 40.0% for dogs and humans, respectively (Table 3).

The mean scores were 1.48 and 0.65 for dogs and humans, respectively, and the difference was highly significant (*p* < 0.001).

## 4. Discussion

In this study, we report a particular begging strategy used by free-ranging Andean dogs. Begging strategies by animals are predominantly based on active behaviors used to obtain food from people. The dogs described in this study used a passive strategy, waiting quietly for the arrival of food. They mainly lay alone where the hairpin bends were formed because of steep slopes, which required vehicles to slow down significantly, thus making it easier for people to throw chunks of bread at the dogs.

Free-ranging dogs are often persecuted by humans since they pose sanitary and hygienic issues [28]. On the other hand, they can also be welcomed and fed on a voluntary basis, as in the case of this study. Dogs are highly skilled at identifying reliable humans and adapting their behaviors to human habits [29]. This explains the particular strategy of begging described in the current study. Free-ranging dogs have a generalist diet and can display a significant range of feeding behaviors [30]. For instance, they can live in proximity to human settlements as scavengers looking for human waste [30,31]. In some cases, they have also been observed to hunt [32,33]. Moreover, begging behavior is also an important part of their feeding strategies [34,35] and in some contexts may become the main method of survival [36]. However, the mode of begging we describe in this study has never been reported before.

It is difficult to explain the begging strategy of dogs based only on instinctual behavior. Dogs clearly learn that if they are alone, they receive all the provided food without needing to fight or share. Moreover, the highly frequent presence of the dogs at the external border of bends, or any place where they are easily seen from afar, confirms that dogs seem to have learned that those positions are the best places to receive food.

The same phenomenon is seen on another road in the same country and is discussed in a technical article on sanitary problems in Bolivia [37]: “*A situation revealed everywhere, dogs included, from this the title, because from Sucre to La Palma (40 km along the Andean precordillera) there is a provincial road where begging dogs are sitting about 500 meters from each other. They are of different breeds but all of them have the common trait of extreme skinniness. Lonely, they wait for the food thrown to them from the few vehicles crossing through all of them defended their territory and we have seen, coming back after 8 h, that they were still there.*” The indicated distance of 500 m is similar to our data more analytically reported in the current paper, which was 1.5–2.0 km as a mean, but only about 250 m when the winding road made the conditions very favorable to begging. 

Free-ranging dogs tend to form packs [38,39], thus the solitary behavior observed in the current study could appear unusual. It should be considered that the number of animals in packs are related to external factors, such as food availability and human persecution [40]. The less food is available, the lower the number of dogs in packs, with lone dogs observed on many occasions [35,39,40,41] (Sen Majumder et al. 2014; Pal et al. 1998). Andean dogs may have learned that breaking with their strong inherited instincts to live in packs provides the advantage of receiving all the food offered without any competition. We do not have an estimate of the potential resources in the location of this study, and thus we are not sure this solitary begging style of Andean dogs was dictated by a paucity of resources, although it is a possible reason. However, we have noted in the villages that some of the solitary dogs observed on the road reunite in packs in the late afternoon (Figure 1D). Therefore, it is also possible that the dogs separate from their pack only during their begging activities as a sort of fission–fusion phenomenon, which is a common social organization in mammals [42]. Fission–fusion groups are well-known in nature. Several factors can regulate the formation of social groups and their separation. The availability of food and the presence of predators seem to be the most important; however, the anthropic factor is an emerging factor that affects the social organization of different species [42], as in the case of the dogs described in this work.

Although the sample size of the recorded humans was limited, we are tempted to compare the begging strategy of dogs and humans. The latter placed themselves in positions with lower probabilities of receiving frequent gifts than dogs. It seems that dogs are more aware of the better positions where they are more easily seen as vehicles must slow down, while humans are not. In addition, humans were more often seen in groups, thus forcing them to share any gains. In fact, the score for dog earnings was almost twice as high as that of humans. However, although this could suggest the presumptive superiority of dogs, it must be underlined that our data is not enough to support such a conclusion. Indeed, we did not measure the actual amounts of money and food received by humans and dogs, nor we can compare the effective values of the gains received by the two. It should also be underlined that the ethical or otherwise complex human social experience strongly affects people’s behavior. The tendency to aggregate is probably stronger in children than in dogs in this specific context. On the other hand, it is also possible that the presence of elderly humans makes it difficult for them to travel long stretches of the road to reach locations more favorable for begging. What is certain is that the two species prefer different strategies of begging.

## 5. Conclusions

Our study provides the first scientific evidence of the particular begging strategy of Andean dogs, which is locally learned. Dogs begged mainly alone, which is the best strategy for avoiding conflicts or sharing food and could potentially be a better one than that used by humans. In addition, only considerably more research of this area would show whether a hierarchy among the dogs exists, allowing the stronger animals to take the better places. Thus, further studies on this topic are needed to identify the mechanisms underlying the begging of dogs and how this behavior can affect the social organization of packs.

## Figures and Tables

**Table 1:** Scores attributed to the observed typologies.

Position	Specificities	Score
External border of the bend	Vehicles must slow down, and the subject is highly visible	2
Internal border of the bend (a)	Vehicles must slow down, and the subject is highly visible at bends not hidden by the mountain’s slope	2
Internal border of the bend (b)	Vehicles must slow down, but the subject is not visible from afar since it is hidden by the mountain’s slope	0
Straight road	The vehicles move quickly, but the subject is clearly visible	1

**Table 2:** Location of dogs and people in the morning and afternoon.

Species	Time	External	Internal (Open)	Internal (Close)	Total Bends	Straight Road
Dogs	Morning	16	6	1	23	14
	Afternoon	36	4	1	41	31
	Total	52	10	2	64	45
Humans	Morning	3	4	1	8	3
	Afternoon	11	2	1	14	8
	Total	14	6	2	22	11

**Table 3:** Location of dogs and people.

Species	External Blind Bends	Internal Blind Bends ^1^	External Open Bends	Internal Open Bends
Dogs	21	2 (8.7%)	31	10 (24.4%)
Humans	3	2 (40.0%)	11	6 (35.3%)

^1^ The localization in the internal part of the blind bends is considered wrong since the subjects are seen too late, thus reducing the probability of receiving food or charity. Both locations are correct in the open bends.
